# Development and validation of the *Short Professional Quality of Life Scale* based on versions IV and V of the *Professional Quality of Life Scale*

**DOI:** 10.1186/s12955-020-01618-3

**Published:** 2020-11-11

**Authors:** Laura Galiana, Amparo Oliver, Fernanda Arena, Gustavo De Simone, José M. Tomás, Gabriel Vidal-Blanco, Inmaculada Muñoz-Martínez, Noemí Sansó

**Affiliations:** 1 Department of Methodology for the Behavioral Sciences, University of Valencia, Av. Blasco Ibañez, 21, 46010 Valencia, Spain; 2 Pós-doutoranda pelo Programa de Pós-Graduação em Serviço Social, Escola de Humanidades, pela Pontifícia Universidade Católica do Rio Grande do Sul (PUCRS), Porto Alegre, Brazil; 3 Pallium Latinoamérica, Buenos Aires, Argentina; 4 Department of Nursing and Podology, University of Valencia, Valencia, Spain; 5 Virgen de La Salud Hospital, Toledo, Spain; 6 Department of Nursing and Physiotherapy, Balearic Islands Health Research Institute IDISBA, University of the Balearic Islands, Palma, Spain

**Keywords:** Compassion fatigue, Professional burnout, Occupational stress, Validation study, Health personnel

## Abstract

**Background:**

This research presents a short version of the *Professional Quality of Life* (ProQOL) scale, one of the most frequently used questionnaires in the arena of applied healthcare investigation. It measures burnout (BO), compassion fatigue (CF), and compassion satisfaction (CS).

**Methods:**

A 9-item version of the ProQOL was developed. In Study 1, this short version, which used items from version IV of the ProQOL, was administered to 817 palliative care professionals from Spain, Argentina, and Brazil. In Study 2, the same nine items, but this time from version V of the ProQOL, were administered to 296 Spanish palliative care professionals.

**Results:**

Study 1: The *Short ProQOL* showed an adequate internal structure, and invariance across the countries studied (*χ*^*2*^(106) = 185.620 (*p* < 0.001), CFI = .929, RMSEA = 0.058 [0.044, 0.072], SRMR = 0.081). Argentinians showed higher levels of BO (mean difference = 0.172, *p* = 0.042, *Cohen’s d* = 0.168), whereas Brazilians showed higher levels of CF (Mean difference = 0.384, *p* = 0.002, *Cohen’s d* = 0.352). Study 2: the Short ProQOL again showed adequate internal structure and reliability (*χ*^2^(24) = 134.504 (*p* < 0.001); CFI = 0.953; RMSEA = 0.126 [0.106, 0.147]; SRMR = 0.063), and was related to coping with death, self-compassion, and self-care.

**Conclusions:**

The *Short ProQOL* could help facilitate the application of harmonizing measurements and its use for cross-cultural comparisons and occupational health monitoring was satisfactory.

## Introduction

In some healthcare areas, professionals are vulnerable to the negative effects of the helping relationship such as compassion fatigue (CF) and burnout (BO), which affect their Professional Quality of Life. For example, Coetzee and Klopper [1] already noted in their work how a prolonged, continuous and intense contact with patient could lead compassion fatigue. In this same line, Dasan et al. [2] argued that organizational factors, such as resources, and individual factors, such as personality and coping strategies, could also favor the emergence of compassion fatigue. Prevalence rates of such phenomena range from 7.3% to 40% for high compassion fatigue in intensive care units [3], and around 60% for medium compassion fatigue in palliative care professionals [4]. In the case of burnout, the rates vary from 14.0% to 70.1% in the case of intensive care units [3], and around 30% of palliative care professionals have shown medium levels of burnout [4]. However, it is at times such as the COVID-19 health crisis, that the risk of suffering these negative processes tends to increase even more. The COVID-19 pandemic has increased pressure on health systems, forcing staff to make critical decisions in environments with multiple adverse conditions. Combined with the fact that their patients often have no therapeutic options, healthcare workers usually end up experiencing a consequent sense of failure. Over extended periods, this situation can lead health personnel to experience acute stress reactions from emotional overload, as well as other affective pathologies or psychosomatic responses. Thus, this health crisis is visibilizing a problem that is already occurring with different degrees of intensity and in different contexts. Understanding the impact that health emergencies have on the quality of care that professionals provide, as well as on their own well-being, requires the availability of valid and easy to apply cross-cultural comparison tools which allow the early identification of CF and BO and the calculation of its incidence. Thus, in the following research we aimed to test a short, 9-item version of the *Professional Quality of Life* (ProQOL) scale, based upon items from its versions IV and V.

As pointed out in the work by Stanton et al. [5], “the multivariate nature of modern organizational research, the apparent ‘survey fatigue’ of organizational members, and new demands to present survey materials online make shortened but psychometrically sound measures of organizationally relevant constructs worthwhile” (p. 168). Therefore, we developed a brief questionnaire based on the ProQOL scale which has been in use since 1995 and has been revised several times, with version V being the current one. In the present study, we used both versions IV and V of the ProQOL. There are hardly any differences between these versions, except for a few wording changes designed to improve their understanding. In Study 1, we used items from version IV of the ProQOL to examine the construct validity, measure invariance across three countries, and the reliability of this new short version. In Study 2, we used version V of the ProQOL to provide more evidence for the usefulness of the short version of this scale and to relate the new short ProQOL with traditional variables related to the quality of life of professionals.

## Background

BO is a syndrome that can be experienced by human services employees in stressful situations [6], with 60% reporting it during their careers [7]. Healthcare professionals are especially vulnerable to BO because their work context is characterized by high-risk decisions, dealing with the public, and expectations of compassion and sensitivity [8]. However, studies have shown that BO alone does not explain professionals’ emotional problems from working with individuals who are suffering or are in pain [9, 10]. In this context, CF, defined as the negative effects of working with traumatized people [11] has received increasing attention in recent years. CF focuses specifically on the chronic worry and tension produced by continued exposure to traumatized individuals [9]. Research on CF has also defined its opposite or converse effect, compassion satisfaction (CS). CS takes place when exposure to traumatic events produces gratification [12] from the joy that comes from helping others [10].

Specifically in healthcare professionals, lower levels of CF and BO have been related to a holistic practice of self-care [13], mindfulness [13], self-compassion [14], empathy [14], or coping with death [13], and CS has been positively related to these variables. For example, Sansó et al. [13] found significant, medium-sized relationships between coping with death and BO, CF, and CS (*β* = − 0.29, *p* < 0.01; *β* = − 0.28, *p* < 0.01; and *β* = 0.33, *p* < 0.01, respectively). In turn, Galiana et al. [14], found relationships between the dimensions of physical, psychosocial, and social self-care, and the three components of Professional Quality of Life. These were higher for the social self-care dimension, ranging from *r* = − 0.27 (*p* < 0.01) to *r* = 0.38 (*p* < 0.01), with lower values for psychological self-care, ranging from *r* = − 0.12 (*p* < 0.05) to *r* = − 0.37 (*p* < 0.01), and physical self-care, ranging from *r* = − 0.12 (*p* < 0.05) to *r* = − 0.22 (*p* < 0.01). Regarding self-compassion, Duarte et al. [15] reported correlations between self-compassion and CS of *r* = 0.32 (*p* < 0.01), of *r* = − 0.44 (*p* < 0.01) with BO, and *r* = − 0.30 (*p* < 0.01) with CS.

Various metrics have been developed to assess BO, CF, and/or CS. For instance, the review carried out by Bride et al. [11] examined six different CF scales to assess the different domains of CF. However, among the reviewed instruments, only the ProQOL scale assessed all the aforementioned constructs [16, 17]. The ProQOL is the most commonly used method for measuring the negative and positive effects of helping others who experience suffering and trauma. This tool assesses professionals’ quality of life via three subscales, the CS, CF, and BO, with each dimension being represented by 10 items. The ProQOL has been widely used across populations, with evidence gathered from informal caregivers [18], professionals working with people with intellectual disabilities [19], social workers [20], or health professionals [13, 21] in several countries, including Australia [21], Brazil [4], Canada [21], China [22], Israel [23], Spain [4], and the United States [24].

Despite its widespread use, evidence on the psychometric properties of the ProQOL is still scarce, with only five studies focusing on its validity. In 2015, Dang et al. [22] studied the reliability and validity of the ProQOL among Chinese governmental staff in areas stricken by the Wenchuan earthquake. They found reliability problems for the BO dimension and a poor fit for confirmatory factor analysis (CFA) model testing for construct validity. Similar results were found by Samson et al. [23] in a sample of Hebrew healthcare providers and found poor reliability results for the BO and CS subscales. However, because they used an exploratory approach, reliability estimates were calculated using a three-factor solution which did not comprise the original items. In 2017, Galiana et al. [4] studied the ProQOL structure in two samples of palliative care professionals from Spain and Brazil and were unable to find significant CFA factor loadings for some items on the BO and CF subscales in either sample, as well as poor reliability estimates for the BO dimension. Finally, and most recently, Hemsworth et al. [21] studied the validity and reliability of the ProQOL in three samples: Australian nurses, Canadian nurses, and Canadian palliative caregivers. They found reliability problems with the BO dimension and in the construct validity when they tested separate CFAs for each subscale in each sample. Problems with the model fit were found in the three models estimated for the BO subscale and in the residual indices of two of the three models estimated for the CF subscale. Finally, Heritage et al. [25] used Rasch analysis to examine the ProQOL in a sample of 1,615 Australian nurses and found a two-factor structure with the CS and CF dimensions, and advocated for a 21-item version which removed items from the BO and CF dimensions.

The availability of a tool that allows Professional Quality of Life to be monitored is of special importance among palliative care professionals such as physicians or nurses who work in environments with a high emotional demand requiring them to face death, loss, and grief on a daily basis. Of note, the quality of life of professionals not only affects the professionals themselves but is also required for quality of patient care. Such a tool should also meet certain criteria. First, the brevity of tools available to health managers and institutions to measure the quality of life of the professionals they oversee (in order to screen and detect potential problems, i.e., prevent BO and CF and enhancing their CS) is especially important. The conditions of working in healthcare contexts (and outcomes to be measured) mean that asking these professionals to answer long questionnaires further increases their workload and reduces their already valuable time. Indeed, as noted by Stanton et al. “longer surveys take more time to complete, tend to have more missing data, and have higher refusal rates than short surveys” [5] (p. 167). Second, the scale should be reliable and valid; we used the original long form of the ProQOL which has several psychometric problems, but only for some of its items. Third, the tool should provide measurement invariance data. As explained by Schmitt and Ali [26], scientists interested in measurement variance are concerned about the reliability and validity of measurement instruments used in different groups and situations. With increased globalization, the applicability of different measurement instruments in various cultures and multicultural and multilingual contexts is of interest both to companies and other institutions. As stated by Schmitt and Ali, “Differences (e.g., in culture, in language) in the populations being measured necessitate examining the degree to which the instrument measures the same construct across these groups” [26] (p. 327). This is to guarantee that individuals with the same construct level receive the same scores, regardless of their group membership. Considering the aforementioned, this study presents a brief measurement tool (the *Short ProQOL*) for assessing quality of life among professionals which is based on versions IV and V of the original ProQOL scale.

## Methods

### Development of the *Short Professional Quality of Life* scale

Based on previous research, we retained the three best items from each dimension or subscale (for item content, see Table 1). For BO, we chose items 10, 19, and 21. According to Galiana et al., these items had the highest factor loadings in the Spanish version [4], except for item 8 which had a slightly higher loading than item 19 (0.639 for item 8 and 0.614 for item 19). However, because item 8 did not work very well in the Brazilian sample (0.374), and items 10, 19, and 21 had the highest factor loadings, we retained the latter structure. Samson et al. offered additional evidence for item 19 by retaining this item as a BO but eliminating item 8 [23]. Moreover, both Hemsworth et al. [21] and the 21-item solution defended by Heritage et al. [25] also provided evidence for the goodness-of-fit of the three items we chose. Additionally, theoretically, these items were consistent with Stamm’s definition of BO in association with feelings of depletion and difficulties in dealing with work (item 10), exhaustion (item 19), and a high workload (item 21) [27].

**Table 1:** Content of the items on the *Short Professional Quality of Life Scale*

Dimension	Original item number	Item content	Short ProQOL item number
CF	9	I think I might have been ‘infected’ by the traumatic stress of those I help (version IV) I think that I might have been affected by the traumatic stress of those I help (version V)	1
BO	10	I feel trapped in my work as a helper (version IV) I feel trapped by my job as a helper (version V)	2
CS	12	I like my work as a helper (versions IV and V)	3
CF	13	I feel depressed as a result of my work as a helper (version IV) I feel depressed as because of the traumatic experiences of the people I help (version V)	4
CS	18	My work makes me feel satisfied (versions IV and V)	5
BO	19	I feel exhausted because of my work as a helper (version IV) I feel worn out because of my work as a helper (version V)	6
BO	21	I feel overwhelmed by the amount of work or the size of the work load I have to deal with (version IV) I feel overwhelmed because the size of my workload seems endless (version V)	7
CF	25	As a result of my helping, I have intrusive, frightening thoughts (versions IV and V)	8
CS	30	I am happy that I chose to do this work (versions IV and V)	9

*BO *burnout, *CF* compassion fatigue, *CS* compassion satisfaction

For CF, again we chose the best items reported by Galiana et al.—items 9, 13, and 25 [4] which were also recently shown to work adequately in samples from Australia and Canada [21], and were retained as CF items both by Samson et al. [23] and Heritage et al. [25]. These items were also representative of the CF construct from a theoretical approach because they include items that specifically measure the traumatic experiences of others (items 9 and 13) and of experiencing symptoms that mimic those observed in the traumatized individuals such as intrusive, frightening thoughts (item 25).

Finally, also based on the results from Galiana et al., we retained items 12, 18, and 30 for CS [4]. Moreover, no problems with the chosen items were reported by Hemsworth et al. [21], Samson et al. [23], or Heritage et al. [25]. As defined by Stamm [27], CS refers to the pleasure derived from being able to do the work, specifically the pleasure of helping others through one’s work. The three items included in the *Short ProQOL* covered this definition of CS by measuring satisfaction with one’s work in general (items 18 and 30) and specifically with helping others (item 12).

### Study 1

#### Design

Three cross-sectional surveys of Spanish, Argentinian, and Brazilian palliative care professionals were carried out. Professionals were sampled using a secure and anonymous online platform (SurveyMonkey) and were encouraged to participate by the Spanish Society for Palliative Care (SECPAL), the Brazilian National Academy of Palliative Care, and the Pallium Latin-American Institute. Participation was voluntary and required the respondents’ informed consent. For inclusion, the participants had to be a healthcare professional (physician, nurse, psychologist, nursing assistant, social worker, or other), who currently cared for patients at the end of their lives, but not necessarily in palliative care settings. As sample cut-off criteria to determine the sample size we used the Monte Carlo data simulation study carried out by Wolf et al. [28]. According to their results (and expecting standardized factorial loadings of 0.65) the minimum sample size for a three-factor CFA model with three indicators per factor would be *N* = 220. Thus, we tried to recruit a suitably large sample size in the three countries included in this study.

#### Participants

*Spanish sample* 385 participants answered the survey. The mean age was 46.8 years (*SD* = 8.87). and 77.55% were women; 40.3% were physicians, 33.1% nurses, 14.2% psychologists, 4.8% nursing assistants, 4.0% social workers, and 0.8% had other professions.

*Argentinian sample* 273 palliative care professionals participated; their mean age was 43.41 years (*SD* = 9.69) and 80.8% were women; 51.5% were physicians, 16.3% psychologists, 14.8% nurses, 8.0% social workers, 1.5% occupational therapists, 1.2% nursing assistants, and 6.8% had other professions.

*Brazilian sample* 161 professionals participated and had a mean age of 37.22 years (*SD* = 11.1); 88.7% were women and 21.1% were physicians, 19.3% nurses, 24.8% psychologists, 5% nursing assistants, 11.8% social workers, and 18% had other professions.

There were statistically significant differences among countries in terms of mean age (*F*(2.786) = 54.589, *p* < 0.001, *η*^2^ = 0.12), sex (*χ*^2^(2) = 8.674, *p* = 0.013, Cramer’s *V* = 0.104), and profession (*χ*^2^(10) = 89.331, *p* < 0.001, Cramer’s *V* = 0.233) distribution across samples.

#### Measurement instruments

This survey included demographic data and a battery of tests designed to measure CS, CF, BO, awareness, coping with death, and specific training, among others. For the purposes of this study, nine items from version IV of the ProQOL [16] in its Spanish and Brazilian Portuguese official versions [4], were used.

#### Data analyses

Statistical analyses included a series of confirmatory factor analyses (CFAs), followed by a standard measurement of invariance routine. CFA is used to explicitly test a priori hypotheses about relationships between observed variables and latent variables or factors [29] and is usually the analysis of choice for refining measurement instruments and evaluating factor invariance across groups [30]. In this work we specified and tested the a priori structure of three correlated factors, including BO, CF, and CS, as in the original ProQOL, in each country. Items 10, 19, and 21 were explained by the BO factor; items 9, 13, and 25 were explained by the CF factor; and items 12, 18, and 30 loaded into the CS factor.

After gathering evidence about the adequacy of this model, we tested the measurement invariance of the factor loadings, intercepts, correlations, and means, as recommended by Thompson and Green [31] and van de Schoot et al. [32]. First, the configural model was tested, to estimate the three-factor structure in the three samples and the goodness-of-fit of this baseline model was compared to that of the other models. Second, we tested metric or weak invariance. The metric invariance model constrains factor loadings to be the same across samples (countries). Third, scalar or strong invariance was tested; the scalar model constrains the intercepts across samples, so that the same estimates for the intercepts would hold for all three samples. Fourth, correlations among factors were constrained, to test whether the relationships among BO, CF, and CS were the same across countries. Finally, latent means across the countries were also constrained.

To assess the model fit, both for single-sample and multi-group models, we used the chi-squared, comparative fit index (CFI), standardized root mean square residual (SRMR), and root mean square error of approximation (RMSEA) fit estimators. A CFI above 0.90 (or, better, exceeding 0.95) and an SRMR or RMSEA below 0.08 (or better, below 0.05) indicated a good fit. However, RMSEA has shown p3oor performance in structural models with low degrees of freedom and in small sample sizes [33, 34]. Indeed, we offer another index for error measurement in the model, the standardized root mean squared residual (SRMR). The SRMR has been defined as “the most sensitive index to models with misspecified factor covariance(s) or latent structure” (p. 424) [35].

Multi-group models were also comparatively assessed. Chi-squared differences tests are usually used for this purpose, leading to the retention of the most parsimonious (in this case, constrained) model when no significant differences between the chi-squares are detected. Given that this approach has been criticized for being too powerful since it can detect even meaningless differences [36], we also used differences between the CFIs of the models tested. Differences of 0.05 or less between two CFIs [37] or of 0.01 or less [36] were considered negligible, and the most parsimonious or constrained model was retained. All the models were tested with MPLUS 8 software [38], and we used a maximum likelihood with robust corrections (MLR) estimation method, given the multivariate non-normality of the data.

Evidence of reliability was also gathered using the composite reliability index (CRI), an index offered in the structural equation model framework and preferred for its robustness [39]. Finally, evidence of overlapping variance with the full form of the ProQOL was also calculated by calculating Pearson correlations among the dimensions of the original versus the short version of the ProQOL.

### Study 2

#### Design

A second cross-sectional survey of Spanish palliative care professionals was conducted during January–February 2020. Professionals were sampled and encouraged to participate again through the SECPAL using same procedure described for Study 1. Participants were sampled from their member lists and were asked to complete an online survey using SurveyMonkey. Participation was voluntary and required the respondents’ informed consent and the same inclusion criteria described in Study 1 were used. The minimum sample size was fixed at *n* = 220, following the results of Wol et al. [28].

#### Participants

The sample comprised 296 palliative care professionals with a mean age of 43.9 years (*SD* = 10.15). A total of 77.40% were women, and 31.8% were physicians, 44.2% nurses, 8.6% psychologists, 4.5% nursing assistants, 5.8% social workers, and 5.1% had other professions.

#### Measurement instruments

This survey included demographic data and a battery of tests designed to measure CS, CF, BO, coping with death, and self-compassion. For the purposes of this study, nine items were used from version V of the ProQOL [17] (see Table 1). We used the backward and forward translation process; first, the scale was translated into Spanish by a professional native; it was then translated back into English by another native professional and no differences were found. No changes were made after review of the Spanish version by the authors, and academics and clinicians expert in Professional Quality of Life and end of life care. In addition, we used:

- The *Coping with Death Scale, Short Version* in its Spanish version [40], comprising nine items and which assesses a general factor of coping with death; its estimate of reliability was 0.858.
- The *Self-Compassion Scale* [41] in its Spanish version [42], comprising 12 items which assess positive and negative self-compassion, with estimates of reliability of 0.785 and 0.824, respectively.
- The *Professional Self-Care Scale* originally developed in Spanish [14], comprising nine items and which assesses the physical, inner, and social dimensions of self-care among professionals; its reliability estimations were 0.737, 0.814, and 0.563, respectively.

#### Data analyses

Statistical analyses included CFA to assess the same three correlated factors, including BO, CF, and CS, tested in Study 1. To assess the model fit we used the same indices as in Study 1. The CRI was also used to gather evidence of reliability. Finally, we examined the relationships with other tests by estimating the correlations between the coping with death, and positive and negative self-compassion dimensions of the *Short ProQOL*.

## Results

### Study 1

The CFA of the *Short ProQOL* tested in the three samples showed an adequate fit, except for the RMSEA, which presented values higher than expected (see Table 2). However, based on Kenny et al.’s results [43], the overall fit of the model in the three samples was considered good.

**Table 2:** Confirmatory factor analysis and set of nested models to test for measurement invariance, Study 1

	*χ*^*2*^	*df*	*p*	CFI	RMSEA	RMSEA CI	SRMR	Δ*χ*^*2*^	Δ*df*	*p*	ΔCFI
CFA in Spain	47.849	24	0.002	0.950	0.055	[0.032, 0.078]	0.047	–	–	–	–
CFA in Argentina	54.956	24	< 0.001	0.913	0.073	[0.048, 0.099]	0.051	–	–	–	–
CFA in Brazil	43.219	24	0.009	0.929	0.089	[0.044, 0.131]	0.069	–	–	–	–
Configural invariance	145.836	72	< 0.001	0.934	0.068	[0.052, 0.084]	0.052	–	–	–	–
Metric invariance	147.267	84	< 0.001	0.943	0.058	[0.042, 0.073]	0.059	4.597	12	0.970	0.009
Scalar invariance	178.921	96	< 0.001	0.926	0.062	[0.048, 0.076]	0.062	33.427	24	0.095	− 0.008
Scalar invariance with equal correlations	182.405	102	< 0.001	0.928	0.059	[0.045, 0.073]	0.082	37.674	30	0.158	− 0.006
Constrained latent means	201.055	108	< 0.001	0.917	0.062	[0.049, 0.075]	0.082	55.664	36	0.019	− 0.017
Unconstrained latent means	185.620	106	< 0.001	0.929	0.058	[0.044, 0.072]	0.081	40.797	34	0.196	− 0.005

*RMSEA CI *90% RMSEA confidence interval

Regarding the invariance, according to the CFI, RMSEA, and SRMR, the configural model fitted the data adequately and so it was retained as the baseline model. When we tested the metric invariance, no statistically significant differences were found between the chi squares and the CFI improved, providing evidence of metric invariance. The scalar invariance test showed no statistically significant differences between the chi squares and a trivial decrease in the CFI. When the correlations among factors were constrained, there were no statistically significant differences or substantial decreases in the CFI, suggesting the equality of the factor relationships across the countries tested. Given that the *Short ProQOL* was found to be an invariant metric, the mean comparisons were constrained. In this case, we found significant differences between the chi-squares and a significant decrease in the CFI. Specifically, statistically significant differences were found between the Spanish and Argentinian BO means, and between the Spanish and Brazilian CF means, with modification indices recommending freeing these equalities. Thus, a new model was estimated in which two latent mean estimates were freed. This latest model was retained as the most parsimonious model; the fit indices are shown in Table 2. Unstandardized and standardized factor loadings and intercepts of the retained model are presented in Table 3.

**Table 3:** Unstandardized and standardized factor loadings and intercept thresholds for the most parsimonious model, the scalar invariant model with equal correlations and unconstrained latent means, tested in the invariance routine of Study 1

Items	Factor loadings				Intercept thresholds
	UN	ST Spain	ST Argentina	ST Brazil	
9	0.446	0.566	0.562	0.446	0.816
10	0.745	0.634	0.550	0.776	1.269
12	0.347	0.521	0.464	0.574	4.635
13	0.572	0.626	0.649	0.568	0.775
18	0.636	0.796	0.775	0.653	4.146
19	0.887	0.780	0.756	0.862	1.782
21	0.931	0.775	0.769	0.889	1.756
25	0.639	0.751	0.703	0.601	0.728
30	0.600	0.764	0.707	0.838	4.465

*UN* unstandardized estimates, *ST *standardized estimates

Regarding the levels of BO, CF, and CS, and when latent means were compared after the invariance routine procedure, our results revealed equal levels of CS across countries but differences in BO and CF. Compared to the Spanish and Brazilian professionals, Argentinian professionals showed higher levels of BO (mean difference = 0.172, standard error = 0.085, *p* = 0.042, *Cohen’s d* = 0.168). Compared to Spanish and Argentinian professionals, Brazilian professionals showed higher levels of CF (mean difference = 0.384, standard error = 0.122, *p* = 0.002, *Cohen’s d* = 0.352). The reliability estimates were adequate, with values of 0.810, 0.763, and 0.737 for BO, CF, and CS, respectively. Finally, the correlations among the long and short versions of the ProQOL were 0.72, 0.78, and 0.75 for BO in Spanish, Argentinian, and Brazilian samples, respectively; 0.83, 0.81, and 0.84 for CF; and 0.87, 0.82, and 0.87 for CS, and these correlations were significant in all cases (*p* < 0.001).

### Study 2

**Fig. 1:**
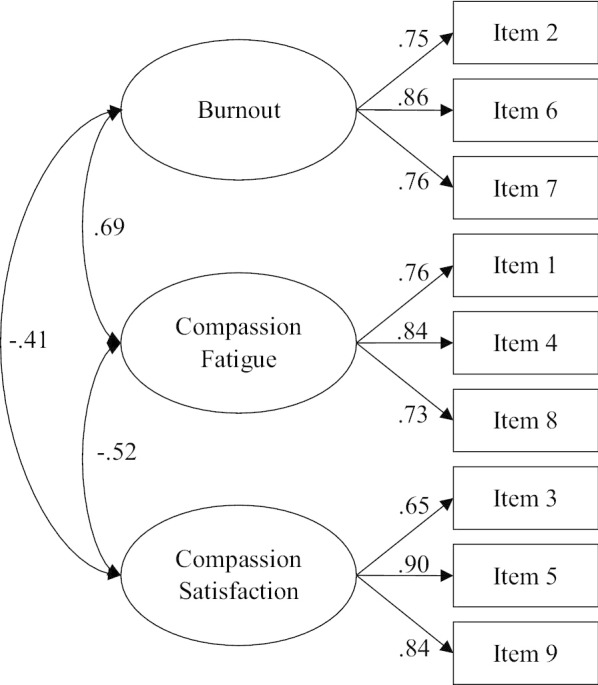
Analytical fit for the *Short ProQOL*, Study 2. *Notes* For the original ProQOL numbers, see Table 1. For the sake of clarity, standard errors are not shown

The three correlated factors tested a priori in Study 1 were analyzed in the nine items of version V of the ProQOL in Study 2. The CFA results were adequate (*χ*^2^(24) = 134.504 (*p* < 0.001); CFI = 0.953; RMSEA = 0.126 [0.106, 0.147]; SRMR = 0.063), except for the RMSEA. However, this index has shown poor performance in structural models with low degrees of freedom [38], and so we considered the fit of this model to be adequate. All the factor loadings for the analytical fit were high and statistically significant and ranged from 0.650 to 0.896 (see Fig. 1).

Reliability estimates were also adequate; the CRI estimate for BO was 0.834, 0.821 for CF, and 0.843 for CS. Finally, we studied the relationship between the dimensions of the *Short ProQOL* with other variables (Table 4). CS was positively related to coping with death, positive self-compassion, and the three dimensions of self-care, and was negatively related to negative self-compassion. In turn, BO and CF were positively related to negative self-compassion and negatively related to coping with death, positive self-compassion, and the dimensions of self-care.

**Table 4:** Relationships between the *Short ProQOL* dimensions with coping with death, self-compassion, and self-care, in Study 2

	Coping with death	Positive self-compassion	Negative self-compassion	Physical self-care	Psychological self-care	Social self-care
BO	− 0.161*	− 0.302**	0.364**	− 0.315**	− 0.207**	− 0.333**
CF	− 0.252**	− 0.241**	0.410**	− 0.196**	− 0.186**	− 0.285**
CS	0.179**	0.279**	− 0.248**	0.157*	0.142*	0.362**

*BO* burnout, *CF* compassion fatigue, *CS* compassion satisfaction; *p < 0.050; **p < 0.010

## Discussion

As Stamm et al. [43] recently expressed in relation to self-care for professionals in these times of pandemic, “Our work can be overwhelming. Our challenge is to maintain our resilience so that we can keep doing the work with care, energy, and compassion”. Therefore, straight-forward tools for monitoring Professional Quality of Life created by consensus approaches are more necessary than ever. The aim of this study was to present a new short version of the widely used ProQOL (versions IV and V). Thus, we tested the validity and reliability of the *Short ProQOL* among palliative care professionals from three different countries: Spain, Argentina, and Brazil in two different studies: in a total, more than 1,000 healthcare professionals.

Other short-version studies validated in samples of palliative care professionals and with good psychometric results are discussed in the scientific literature, for example, the *Bugen Scale Of Coping With Death* [40] or the *Swedish Frommelt Attitudes Toward Care of the Dying Scale* [44]. It is worth highlighting the importance of the availability of brief surveys for use in demanding work contexts such as end-of-life care in order to help protect these staff and not overload them with long questionnaires that further increase their burden and stress. Indeed, as already mentioned, an enormous range of conditions and outcomes can be measured in healthcare personnel but having to answer long questionnaires increases the workloads of these professionals and reduces their valuable time. Moreover, the use of long surveys is also associated with higher missing data and refusal rates [5].

To develop the new *Short ProQOL*, we combined information about the previous statistical performance of items from the long-version ProQOL, taking a theoretical approach. This is a common procedure for reducing long scales with problems with their psychometric properties. Previous studies focusing on the properties of the ProQOL reported problems with its reliability and validity, especially in the BO and CF dimensions [4, 21–25]. Hence, we examined the 30 items on the ProQOL scale and retained those with no reported psychometric problems and which were consistent with Stamm’s [27] definition of BO, CF, and CS. Three items from each dimension were retained: items 10, 19, and 21 for BO; 9, 13, and 25 for CF; and 12, 18, and 30 for CS.

In Study 1, the new scale structure with the chosen items was tested in the three different samples (countries) using the short ProQOL version IV. The CFAs showed an adequate fit, thus offering evidence of the *Short ProQOL* construct validity. The structure tested maintained the three dimensions of quality of life among professionals (the BO, CF, and CS) proposed by Stamm [16, 17], but with only three items on each subscale. However, the retained items respected the original meaning of the dimensions, which focused on feelings of depletion and difficulties in dealing with work and a high workload (BO); being affected by others’ traumatic experiences and having symptoms similar to the ones observed in traumatized individuals, especially intrusive and frightening thoughts (CF); and satisfaction derived from work and from helping others (CS).

There was also evidence for the reliability of the *Short ProQOL* based on relevant estimates for the three dimensions or subscales. Compared to the previous results obtained with the long form of the ProQOL, this short version solved the reliability problems in the BO [4, 22] and CF dimensions [23] which had also been identified in previous shortened versions (i.e., [23]). In addition, the CFA model showed an appropriate factorial structure fit, therefore solving fit problems reported in the long version by Samson et al. [20] and Hemsworth et al. [21]. Finally, although the 21-item version presented by Heritage et al. [25] did not report any problems with reliability or validity, it is worth mentioning that these authors conducted their work in only one study of the scale (in an Australian sample). Here we provided evidence of the adequate behavior of the *Short ProQOL* in three different countries, speaking two different languages, and in two different studies (see the results of Study 2).

Once the structure was successfully tested in the three samples, our invariance analysis indicated scalar invariance of the *Short ProQOL* across the samples (countries). Although frequently understudied, measurement invariance is a core issue when making group comparisons when the groups can be understood as different populations (different countries, races, cultures, professions, etc.) [33]. To meaningfully compare relationships across groups, metric invariance is a necessary condition, while for group mean comparisons, scalar invariance is necessary [45]. However, these requirements were not met in most research regarding the ProQOL [24, 46–59]. In our case, because the measurement invariance routine results were successful, we were able to test for mean differences. These results suggested that (1) BO was higher among the Argentinian professionals compared to the Spanish and Brazilian professionals; (2) CF was higher among Brazilians, compared to Spanish and Argentinian professionals; and (3) CS was the same among the three samples. Thus, these results suggest that this short survey using only nine items could be used in further research to capture differences attributable to context.

The differences in the age, sex, and distribution seen in the samples could have been because of different levels of BO and CF in relation to age, sex, or different professions of the participants. However, previous research has shown some controversy regarding differences in professionals’ quality of life in relation to these variables [24, 46–59], and so future research should examine this possible explanation. Several studies have noted that sex is an important variable because women experience more BO than men [43, 44]. However, very few studies have examined sex differences related to CF. Of these, Van Hook and Rothenberg found that, in a sample of 175 child welfare workers (136 male and 28 female) with a variety of assignments, females reported more CF than males, with marginal significance [49]. Furthermore, an investigation conducted with ICU and oncology nurses, concluded that male nurses exhibited significantly higher CS and lower BO and CF than female nurses [50]. Similarly, one study assessing pediatric nurses in the USA [24] and another examining a sample of 1121 mental health professionals [51] concluded that female sex was associated with higher mean CS and CF scores than male sex. They also showed that psychiatrists experienced higher CF than other professionals.

However, another study [52] conducted among a total of 532 clinical social workers and psychologists, concluded that sex had no significant effect on CF. Moreover, results from the study conducted by Mooney et al. [50] showed that CF decreased with years of nursing experience, although this relationship did not fit with the other dimensions. A broader relationship was found in a cross-sectional study of registered nurses working in emergency departments throughout the United States [53]; they obtained significant differences in the Professional Quality of Life results for the CF, BO, and CS dimensions in relation to respondent age, but no statistical significance when comparing the difference between male and female nurses [53]. Additionally, the study conducted by Sprang et al. [54], examined 669 mental health workers and child welfare workers and found that males experienced significantly higher levels of CF compared to females.

Regarding differences among professions, most research indicates that the nursing profession is most affected by BO and CF. For instance, a recent study among professional care providers at Palliative Cancer Care Centers in India showed that nurses and nursing aids had significantly higher BO than the other professionals studied (physicians, social workers, physiotherapists, and pharmacists) [55]. This also fits with previous research indicating that the presence of BO and risk of CF is higher in nurses than in other health professions [56–59]. Therefore, we concluded that the differences in CF and BO observed in our results may have been related to sex, because CF was highest in Brazilian professionals—the group which included the most women. However, the relationship of these dimensions with profession remains unclear, because BO was higher in Argentinian professionals—the group with the most physicians and least nurses, a result which contrasts with the literature we reviewed. Therefore, differences in BO across samples could be because of the effect of the country rather than the profession, and further research will be required to clarify this.

In Study 2, we used version V of the ProQOL for the new short version of the ProQOL; this version differed from version IV in five out of the nine items comprising the Short ProQOL version used in Study 1: the three BO dimension items and two of the CF dimension items. This second study confirmed that the short structure, with adaptations of these items, also showed evidence of an adequate factorial structure with adequate reliability indices and relationships with previous variables such as coping with death, self-compassion [15], and self-care. Competence in coping with death is key both for adequate professional development and Professional Quality of Life. There is also evidence to suggest that the absence of this capacity can involve emotional distress, BO, and CF, while its presence has been related to CS [4, 13, 60]. Moreover, recent research has found an association between higher levels of self-compassion and lower levels of BO and CF, as well as a positive relationship between self-compassion and CS. Indeed, Beaumont et al. [61] gathered evidence for an inverse relationship between self-compassion and BO in a sample of experienced psychotherapists and this same relationship was also found in a sample of nursing professionals [62].

Additionally, self-compassion promotes interpersonal skills and is related to other quality of life determinants among professionals, such as empathy [15, 63]. The proper practice of self-care was a key aspect for maintaining health and Professional Quality of Life [14]. Aukstinaityte and Zajanckauskaite-Staskeviciene [64] were some of the first authors to evaluate this, offering evidence of a negative relationship between self-care and CF which was also supported by evidence from work by Neville and Cole [65]. Moreover, Galiana et al. [14] and Sansó et al. [13] published evidence for the negative relationship between CF and BO, and the positive link between self-care and CS, both directly and indirectly. More recently, Sorenson et al. [66] reviewed published qualitative data and found that self-care was the most significant preventative measure that healthcare professionals had reported taking to protect themselves from developing CF.

Finally, we would like to mention that this study was limited by the relatively small sample sizes we used in the Brazilian and Argentinian cohorts, which could have affected the generalization of our results. Another limitation was that we did not study the content or face validity of the retained items. Although we examined the relationship of the items with Stamm’s original definitions for the dimensions, no specific analyses were conducted to test this. Regarding evidence of overlapping variance with the full form of the survey, we only gathered this data in one study rather than in independent administrations, as recommended in the literature [67]. Another shortcoming of this research was that there were significant differences in mean participant ages, and sex and profession distribution in each of the samples. Thus, these differences could also have been because BO and CF change in relation to age, sex, or different professions. Thus, evidence in this regard from future work would be welcomed.

## Conclusions

The main conclusion of this study was that the new *Short ProQOL* scale provides a robust way to measure the quality of life of professionals. Furthermore, its brevity is not incompatible with improved psychometric properties and, based on relationships with other variables, the results for factorial validity, invariance measurement, reliability, and validity, were also appropriate.

## Data Availability

The datasets used and/or analyzed during the current study are available from the corresponding author upon reasonable request.

## Abbreviations

BO: Burnout
CF: Compassion fatigue
CFA: Confirmatory factor analysis
CFI: Comparative fit index
CRI: Composite reliability index
CS: Compassion satisfaction
ProQOL: *Professional Quality of Life Scale*
RMSEA: Root mean square error of approximation
SECPAL: Spanish Society for Palliative Care
SRMR: Standardized root mean square residual
